# Impact of day care services on physical and cognitive functions in old people with dementia in a medical center in central Taiwan

**DOI:** 10.1186/s12955-021-01806-9

**Published:** 2021-06-24

**Authors:** Cheng-Fu Lin, Jia-Jyun Wu, Yu-Hui Huang, Li-Ying Ju, Shih-Yi Lin, Ying-Chyi Chou, Chu-Sheng Lin

**Affiliations:** 1 Center for Geriatrics and Gerontology, Taichung Veterans General Hospital, 1650 Taiwan Boulevard Sect. 4, Taichung, 40705 Taiwan, ROC; 2 Division of Occupational Medicine, Department of Emergency, Taichung Veterans General Hospital, Taichung, Taiwan, ROC; 3 Department of Family Medicine, China Medical University Hospital, Taichung, Taiwan, ROC; 4 Department of Nursing, Taichung Veterans General Hospital, Taichung, Taiwan, ROC; 5 School of Nursing, Chung Shan Medical University, Taichung, Taiwan, ROC; 6 Department of Business Administration, Center for Healing Environment Administration and Research (HEAR), Tunghai University, No. 1727, Sec. 4, Taiwan Boulevard, Xitun District, Taichung, 40704 Taiwan, ROC

**Keywords:** Day care, Dementia, Family caregiver, Geriatric assessment, Quality of life

## Abstract

**Background:**

Day care service (DCS) provides various activities in a professional environment to meet the old people with functional limitations. However, relatively little is known about the effects of DCS on physical and mental functions.

**Methods:**

This was a retrospective study that we used a comprehensive geriatric assessment to evaluate the changes before and after DCS among participants in a hospital-affiliated geriatric day care center in Taiwan. The burden of the participants’ families was also assessed.

**Results:**

The 18 participants with a median age of 80.9 (interquartile range (IQR) 75.2–86.6 y/o) were enrolled and followed up for 6 months. Based on the clinical dementia rating (CDR), disease stage was very mild in 3 participants, mild in 10, moderate in 3, and severe in 2. The activities of daily living (ADL) scores of the participants improved significantly from 75 (IQR 60.0–80.0) at baseline to 77.5 (IQR 65.0–90.0) at the 6 month (*p* < 0.001), and mini-mental state examination (MMSE) scores from 15 (IQR 11.5–20.0) to 18 (IQR 15.8–24.0) (*p* = 0.026). There was a positive correlation of baseline mini-nutritional assessment-short form score and the 3-level version of the European Quality of Life-5 dimensions utility index with both ADL and MMSE scores at the 6-month follow-up. In addition, the family burden scale was reduced from 22 to 15 (*p* = 0.002).

**Conclusions:**

The physical and cognitive functions in old people with dementia who received DCS were maintained or partially improved, and their families’ stress burden was alleviated.

## Background

Taiwan’s population reached the demographic threshold that defines a society as aged in April 2018, with more than 14 percent of the population aged 65 years or older [1]. Accordingly, the number of people with physical or cognitive disability as well as care needs is expected to increase. In response to the growth of Taiwan’s elderly population, the government launched a long-term care plan, known as long-term care (LTC) version 1.0, in 2008, which provides the elderly and disabled with home nursing, meal provision, and transportation, as well as rehabilitation and respite care services [2]. In 2017, it is estimated that about 113,000 people received long-term care based on an annual report of the Ministry of Welfare in Taiwan [3], and an updated version, LTC version 2.0, was implemented, which additionally included a number of community-based services, such as preventive care in the community, dementia care, discharge planning, and medical home services [4]. The LTC version 2 is intent to provide a more comprehensive integrated care system that will allow older adults and disabled individuals to live at home or in their communities as long as possible. Among the multiple LTC service models, day care service (DCS) is designed to meet the daily living and social needs of adults with functional limitations during the day in a supportive and professionally staffed environment [5]. In addition, non-pharmacological protocols are employed to promote cognitive and everyday practical skills. Several studies have shown that DCS allows disabled old people (e.g., dementia patients) to maintain close contact with their home environment in the community [6]. DCS also delays functional decline and increases quality of life [7]. The utilization of DCS significantly reduced the mortality rate among community-dwelling, frail older people [8], and decreases the risk of hospitalization or admission to nursing homes [7]. Besides, DCS can reduce the time and burden of caregiving, and increase caregivers' life satisfaction [6].

Several surveys have shown that participants and their family in Taiwan were satisfied with DCS [9]. However, the effects of DCS on changes in physical and mental functions have not been studied in detail. The comprehensive geriatric assessment (CGA), which is composed of traditional clinical examinations, functional evaluations, nutritional assessments, and neuropsychological evaluation, is widely used for the evaluation of geriatric conditions, and planning of interventions in the elderly [10]. As functional improvement is crucial in the planning of a patient’s care needs and rehabilitative strategies, the first aim of this study was to investigate any changes in mobility, physical and mental functional status by serial CGAs among patients receiving DCS at a geriatric day center. Second, we aimed to examine whether DCS affected the burden and life satisfaction of the patients’ families.

## Methods

### Participants

In Taiwan, according to LTC version 2, senior citizens 65 years of age or older who are mildly to moderately disabled and adults 55 years of age or older with dementia diagnosis can apply for DCS as long as it can be shown that they are not capable of managing their daily routine to some extent. The severity of disability was evaluated using the clinical frailty scale (CFS), which classifies patients into nine categories based on their dependence on others [11]. The diagnosis of dementia was based on previous medical records, and disease severity was determined by the clinical dementia rating (CDR) [12]. According to the regulations of the Ministry of Health and Welfare, Taiwan, the maximum number of participants who can receive DCS at any given time is decided by area and the number of staff number at each day care center. Therefore, the maximum number allowed is 20 in our day care service. This study was approved by the Institution Review Board of the hospital (Reference Number: CE20010B).

### Study design

This study retrospectively reviewed the serial follow-up data of physical and mental function status in participants who received DCS in a hospital-affiliated day care center in central Taiwan between February 2018 and November 2019. During the study period, a total of 26 participants, who were enrolled consecutively, received DCS at our day care center. Among them, 18 had the follow-up time more than 6 months (5 more than 18 months, 3 between 15 to 18 months, 4 between 12 to 15 months, 3 between 9 to 12 months, and 3 between 6 to 9 months), and 8 less than 6 months. Finally, we analyzed the data in 18 participants with at least 3 serial CGAs (e.g. one baseline and two follow-up). The day care center staff comprised five formal care givers and a leading registered nurse who had received dementia-specific training. Their duties mainly involved general daily services, health programs, and related activities. At admission, general demographic data of the participants, including lifestyle habits, age, gender, body mass index, social, and family histories and medical histories, including diagnosed diseases and medications, were recorded. Moreover, at baseline, and every 3 months thereafter, a Chinese version of CGA was conducted by the registered nurse for each participant to evaluate underlying geriatric problems and changes after DCS [13]. If the participants had medical, functional, and/or social difficulties, they were referred to geriatricians, dietitians, rehabilitation therapists, psychiatrists, or social workers, as appropriate. The various DCS programs were run by the designers with assistance from formal care giver in the day care center, and every activity was provided for the participants together twice per week, and spent approximately 1 h per section. Caregiving burden of the main family caregiver was assessed by the questionnaire at the admission of the participants and after 6-month DCS.

### Assessment

In brief, the components of the CGA, as previously described [10], includes basic personal information (age, gender, history of chronic illness, education, source of referral) and various assessment tools, including the Activities of Daily Living (ADL) scale was evaluated using the Barthel Index, the Lawton Instrumental Activities of Daily Living (IADL) scale, and Mini-Mental State Examination (MMSE). Additionally, a five-item Geriatric Depression Scale (GDS-5) was used to screen for depressive symptoms in the elderly patients with a cut-off score of ≥ 2points for the presence of depressive symptoms. The Mini -Nutritional Assessment-Short Form (MNA-SF) was used to identify older adults who have or are at risk for developing malnutrition with a total score ranging from 0 to 14 [14]. A cut-off of ≥ 12 points is regarded as an indication of being well-nourished, from 8 to 11 points indicates a risk of malnutrition, and < 7 points indicates the person is malnourished. Frailty was defined according to the criteria of the Cardiovascular Health Study Group [15]. Handgrip strength was measured by a dynameter (Smedley's Dynamometer, TTM, Tokyo, Japan), and slowness was measured by a 6-m walking test. Comorbid conditions were measured using the age-adjusted Charlson Comorbidity Index (ACCI), which was a combination of the age equivalence index and the Charlson Comorbidity Index originally included 19 chronic diseases that were weighted based on their association with mortality [16]. The attendance rate was measured by the completion percentage of attending various DCS programs by the participants.

### Intervention

- Reminiscence therapy
  The group leader presented some pictures that would stimulate the elders’ memories, and then listened as the participants talked about them.
- Exercise therapy
  Patients performed resistance and stretching movements for 15–50 min five times per week.
- Cognitive occupational therapy
  Patients were asked to name various different objects, and were taught the appropriate use of different tools.
- Art therapy
  To encourage the use of the fine muscles of the hand, participants were invited to draw.
- Horticultural therapy
  Horticultural therapy consisted of planting plants and creating flower-based decorations.
- Music therapy
  Participants were encouraged to express themselves musically by singing folk songs or other popular songs.

### Measurements of life quality and caregiver burden

Life quality was measured by the 3-level version of European Quality of life-5 dimensions (EQ-5D) questionnaire, which consists of two elements designed for self-completion: the EQ-5D descriptive system and the EQ visual analogue scale (EQ-VAS) [17]. The descriptive system comprises measures of mobility, ability to perform activities of self-care (e.g., washing and dressing), ‘usual’ activities (e.g., work, study, housework, family and leisure activities), and levels of pain/discomfort and anxiety/depression. Each of these dimensions is divided into three levels of perceived problems: Level 1 indicating no problems, Level 2 indicating some problems, and Level 3 indicating extreme problems. The 3-level version of EQ-5D score is mathematically converted to an EQ-5D utility index for analysis, and a higher EQ-5D utility index score indicates a better quality of life. The Chinese version of this questionnaire has been validated in a Taiwanese population [18]. The EQ-VAS is a single index value for health status which records the participants’ self-rated health using a 100-point vertical visual scale ranging from “worst imaginable health state” (0) to “best imaginable health state” (100) [17]. The caregiving burden is self-assessed based on the clinical caregiver’s recollection of stress, and the test includes a total of 14 questions in Chinese [19]. For each item, the family caregiver is asked to respond with one of four selections, which are as follows: never, with a score of zero; rarely, with a score of 1; sometimes, with a score of 2; and quite frequently, with a score of 3. The level of caregiving burden was obtained by aggregating the total scores from these 14 questions. The caregiving burden of each family caregiver based on the total score was classified into three levels, i.e., 0–13 (little or no burden), 14–15 (moderate burden), and 26–42 (severe burden).

### Statistical analyses

Continuous variables were expressed as median and interquartile range (IQR 25–75%). Categorical data were expressed as number and percentages of the total due to the small sample size. Paired comparisons were made using the Wilcoxon signed rank test or Friedman test for continuous variates, and McNemar’s or Cochran’s Q tests for categorical variates during follow-up. Spearman's correlation analysis was used to measure the relationships between various baseline parameters and physical and cognitive function after the 6-month DCS. Statistical analyses were performed using SPSS version 22.0 (SPSS Inc., Chicago, IL, USA). A p value of less than 0.05 was considered to be significant.

## Results

### Participants’ characteristics and functional changes before and after DCS

In total, the median age of the studied participants was 80.9 (IQR 75.2–86.6) years old, with a predominance of women (72.2%), and 72.2% of patients were older than 75 years of age (Table 1). The age-adjusted Charlson comorbidity score was 6 (IQR 4.0–7.0) and the five most common pre-existing comorbidities were hypertension, diabetes, visual, musculoskeletal, and cardiovascular diseases. Based on clinical dementia rating, disease stage was very mild in 3 of the participants, mild in 10, and moderate in 5. Seventeen participants had a CFS level of 5 to 7. The attendance rate was 94.8 (IQR 86.8–99.4). The baseline and 6-month follow-up CGA scores were displayed in Table 2. The ADL scores at baseline, and 6-month follow-up were 75 (IQR 60.0–80.0), and 77.5 (IQR 65.0–90.0), (*p* < 0.001). The MMSE scores at baseline, and 6-month follow-up were 15 (IQR 11.5–20.0), and 18 (IQR 15.8–24.0), respectively (*p* = 0.026). An improvement was observed for ADL and MMSE score, although MNA-SF score, walking speed, and hand grip strength showed no significant difference. The differences between baseline and 6-month follow-up for physical and cognitive function are shown in Table 3. The baseline EQ-5D utility index and MNA-SF score were found to be associated with both ADL and MMSE scores at the 6-month follow-up after DCS.

**Table 1:** Demographic characteristics of the participants

Variable	Total	
Age (years)	80.9	(75.2–86.6)
Age more than 75 y/o (M/F)	4/9	(72.2%)
Gender (n)		
Male	5	(27.8%)
Female	13	(72.2%)
BMI (kg/m^2^)	25.2	
CDR	1	(1.0–2.0)
CFS	5	(5.0–6.0)
Comorbid disease number	4	(2.8–5.0)
Comorbidities		
Hypertension	13	(72.2%)
Diabetes mellitus	7	(38.9%)
Visual disease	6	(33.3%)
Musculoskeletal disease	5	(27.8%)
Cardiovascular diseases	4	(22.2%)
Cerebrovascular accident	2	(11.1%)
Chronic kidney disease	2	(11.1%)
Benign prostatic hyperplasia	2	(11.1%)
Cancer	2	(11.1%)
Parkinson's disease	1	(5.6%)
Others^a^	6	(33.3%)
ACCI	6	(4.0–7.0)
Laboratory data		
WBC (/μL)	6145	(5305.0–7475.0)
Hemoglobin (g/dl)	13.4	(12.8–14.2)
Creatinine (mg/dl)	0.9	(0.7–1.1)
Albumin (g/dl)	4.1	(3.9–4.2)
ALT (U/L)	14.5	(12.6–18.5)
Cholesterol (mg/dl)	86	(74.5–124.2)
Triglycerides (mg/dl)	156	(128.5–185.8)
Fasting plasma glucose (mg/dl)	94.5	(86.8–109.8)
Baseline CGA data		
ADL	75	(60.0–80.0)
IADL	2.5	(1.0–3.0)
MMSE	15	(11.5–20.0)
GDS-5	1	(0.0–1.0)
MNA-SF	12	(11.0–14.0)
Hand grip strength (kg)	15.2	(13.4–21.8)
Walking speed (s)	10.4	(7.2–17.8)
Functional reach test (cm)	17.6	(11.0–24.2)
EQ-5D utility index	0.7	(0.4–1.0)
EQ-VAS	70	(60.0–97.5)
Attendance rate (%)	94.8	(86.8–99.4)

ACCI, Age-adjusted Charlson Comorbidity Index; ADL, activities of daily living; ALT, alanine aminotransferase; BMI, body mass index; CDR, clinical dementia rating; CFS, clinical frailty scale; CGA, comprehensive geriatric assessment; GDS-5, five-item geriatric depression scale; IADL, instrumental activities of daily living; MMSE, mini-mental state examination; MNA-SF, mini nutritional assessment short-form; VAS, visual analogue scale; WBC, white blood cell count

^a^Others were osteoporosis, ear disorder, lung disease, gynecological disease, disorder of lipoprotein metabolism

**Table 2:** CGA at baseline, 3- and 6-month follow up

	Baseline		3-month		6-month		*p* value
	Median	IQR	Median	IQR	Median	IQR	
ADL	75	(60.0–80.0)	80	(68.6–90.0)	77.5	(65.0–90.0)	< 0.001
IADL	2.5	(1.0–3.0)	2	(1.0–3.0)	2	(1.0–3.0)	0.028
MMSE	15	(11.5–20.0)	16.5	(14.8–22.0)	18	(15.8–24.0)	0.026
GDS-5	1	(0–1.0)	1	(0–2.0)	1	(0–1.3)	0.734
MNA-SF	12	(11.0–14.0)	13	(12.0–14.0)	13	(11.8–13.3)	0.352
Hand grip strength (kg)	15.2	(13.4–21.8)	13.4	(12.1–21.4)	15.0	(12.2–21.0)	0.943
Walking speed (s)	10.2	(7.1–17.7)	10.2	(7.9–18.9)	13.8	(6.0–27.6)	0.878
Functional reach test (cm)	17.6	(11.0–24.2)	21.6	(13.7–25.7)	17.3	(12.3–22.0)	0.640
EQ-5D utility index	0.7	(0.4–1.0)	0.6	(0.4–0.7)	0.5	(0.3–0.7)	0.097
EQ-VAS	70	(60.0–97.5)	80	(60.0–89.5)	70	(35.0–80.0)	0.247

ADL, activities of daily living; GDS-5, five-item geriatric depression scale; IADL, instrumental activities of daily living; MMSE, mini-mental state examination; MNA-SF, mini nutritional assessment short-form; VAS, visual analogue scale

**Table 3:** The correlation between baseline parameters and physical and cognitive function at the 6-month follow up

	ADL (6 month)		MMSE (6 month)	
	r_s_	*p* value	r_s_	*p* value
Baseline CGA data				
ADL	0.9	< 0.001	0.3	0.204
IADL	0.6	0.008	0.3	0.315
MMSE	0.4	0.156	0.7	0.001
GDS-5	-0.5	0.036	-0.2	0.376
MNA-SF	0.5	0.047	0.4	0.069
Hand grip strength (kg)	0.1	0.795	0.2	0.401
Walking speed (s)	0.8	0.001	− 0.1	0.686
Functional reach test (cm)	0.7	0.002	0.2	0.452
EQ-5D utility index	0.6	0.005	0.5	0.044
EQ-VAS	-0.1	0.741	− 0.1	0.701

ADL, activities of daily living; CGA, comprehensive geriatric assessment; GDS-5, five-item geriatric depression scale; IADL, instrumental activities of daily living; MMSE, mini-mental state examination; MNA-SF, mini nutritional assessment short-form; VAS, visual analogue scale

### Assessment of quality of life and family caregiver burden

At baseline, participants reported a median EQ-5D utility index of 0.7 (IQR 0.1–1.0), an EQ-VAS score of 70 (IQR 60.0–97.5), and GDS of 1 (IQR 0–1.0) (Table 1). After 6 months of DCS, the EQ-5D utility index was marginally better, although there was no statistically significant change compared with the corresponding baseline scores (Table 2). With respect to the family caregivers’ burden, the score was also decreased after the 6-month follow-up, as shown in Table 4.

**Table 4:** Comparisons of family caregiver burden between baseline and 6-month follow up

Questions	Baseline		6-month		*p* value
	Median	IQR	Median	IQR	
1. Do you feel uncomfortable when you need to take care of him/her while you are ill?	2	(0.5–3.0)	1	(1.0–2.0)	0.160
2. Do you feel exhausted because of taking care of him/her?	2	(1.5–3.0)	2	(1.0–2.0)	0.004
3. How burdened do you feel physically in caring for him/her?	1	(0.5–2.5)	1	(0.5–2.0)	0.190
4. Are you affected by his/her emotion?	1	(0.0–1.5)	1	(0.0–1.0)	0.480
5. Do you feel sleep disturbance because he/she cannot sleep at night?	2	(0.0–2.5)	1	(0.0–1.5)	0.008
6. Do you feel your health has suffered because of your involvement with him/her?	1	(0.0–2.0)	1	(0.0–1.5)	0.059
7. Do you feel strained when you are around him/her?	2	(1.0–2.0)	1	(1.0–2.0)	0.005
8. Do you feel mentally painful when you are around him/her?	2	(0.5–2.0)	1	(0.0–2.0)	0.020
9. Do you feel angry when you are around him/her?	2	(0.5–2.5)	1	(0.0–2.0)	0.020
10. Do you feel that your social life has suffered because you are caring for him/her?	2	(0.5–2.0)	2	(0.0–2.0)	0.279
11. Do you feel uncomfortable about having friends over because of him/her?	2	(0.5–2.0)	1	(0.0–1.5)	0.014
12. Do you feel he/she is dependent on you?	3	(2.0–3.0)	2	(1.0–2.5)	0.005
13. Do you feel that you do not have enough money to take care of him/her?	1	(0.0–2.0)	0	(0.0–1.5)	0.408
14. Do you feel that your income has been affected because of taking care of him/her?	1	(0.0–2.0)	0	(0.0–1.0)	0.063
Total score	22	(11.5–28.5)	15	(9.5–22.0)	0.002

## Discussion

Slowing the progression of functional impairment and maintaining independence are important goals in the care of older people. Thus, DCSs were developed to prevent isolation, depression, and undue cognitive and physical decline among community-dwelling older adults [5, 7, 8]. As such, we investigated whether DCS was beneficial in terms of changes in functional status among Taiwanese participants. In our study, the CGA results showed that there was a significant improvement in physical function and cognition status among participants after receiving 6 months of DCS. In addition, there was a correlation of baseline MNA-SF score and EQ-5D utility index with both ADL and MMSE scores at the 6-month follow-up after DCS.

In Taiwan, DCS can be generally divided into two models according to the different needs and characteristics of the clients [20]. The first is the so-called social care model, which provides socialization, as well as creative and educational activities, but does not include personal care. Therefore, participants in the DCS social model are usually independent. The second is known as the medical care model, which provides medical, nursing, and personal services with physical and occupational rehabilitation, and other types of therapies. In this type, most of the participants are disabled, either physically or mentally. In our hospital-affiliated day care center, we provide a combined service with both hospital and social programs in order to better integrate care provided by the hospital, the community, and the home.

Many studies have shown that DCS is effective at improving participant outcomes, including cognition, behaviors, physical functioning, and overall well-being [21–24]. Consistent with previous reports [23, 24], our study demonstrated that there were significant improvements in physical and cognitive functions in older people with dementia after a 6-month DCS program. For dementia patients, multidomain programs could have additive effects, yielding superior results compared with those achieved with just one activity [25], and such programs could help preserve cognitive function and emotional stability [26]. For example, exercise might facilitate neuroplasticity and prevent hippocampal regression associated with memory loss [27], and may also reduce the risk of injury due to falls [28]. Cognitive occupational therapy can improve cognitive and memory function [27]. Reminiscence therapy encourages patients to share memories with each other, which could help cognitive function and/or improve emotional state [29]. Art‐based therapy is a promising component of dementia care, as shown in a previous study of patients with probable dementia in which art therapy improved behavioral parameters, self‐caring, and social interactions [30]. Group music therapy has been reported to improve short‐term memory, and reduce depression with minimal cost [31]. Moreover, horticultural therapy can provide a sense of stability, improve the quality of sleep, reduce the use of neuropsychological drugs, decrease the occurrence of fall‐related injury, and induce behavioral improvements [32]. Overall, our results support the findings of previous research showing that the multidomain programs in DCS improved mobility and cognition in the dementia participants within a short period.

DCS can also affect the overall wellness of participants, including emotional problems, perceived psychosocial well-being, and positive changes in social support and quality of life through various programs provided via DCS. In our study, there was a marginal improvement of EQ-5D utility index scores after the 6-month DCS. Furthermore, baseline EQ-5D utility index scores, especially measures of mobility and ability to perform activities of self-care domains (data not shown), were predictive of physical and cognitive functions at 6 months. A similar result has been shown in frail older people for whom higher baseline EQ-5D was associated with better maintenance of cognitive decline [33]. It is thought that patients with higher baseline quality-of-life scores may have better functional reserves, and thus would be more likely to benefit from DCS. This finding is important, as it suggests that assessment of severity of each condition in the EQ-5D may be necessary to improve outcomes in dementia participants receiving DCS. Regarding the value of EQ-VAS, it was speculated that at the beginning of DCS (e.g. within 3 months), patients may have be more optimistic, and thought their condition can be better managed in association of with improvement of EQ-VAS scores. However, after 6-months DCS, due to either episodic symptoms or progression of underlying disorders, EQ-VAS values returned to that at baseline. More disease-specific questionnaires for episodic conditions may be necessary to rate quality of life.

In line with previous studies showing a positive impact of nutrition status on the outcomes of physical and cognitive functions in old people with dementia, our study also found a correlation between participants’ baseline MNA-SF score and both ADL and MMSE scores at the 6-month follow-up [34]. The improvement of physical and social activities may lead to beneficial effects on dementia patients’ appetite, eating habits, and patients’ families’ knowledge of nutritional support, which could subsequently enhance patients’ nutritional status [35]. However, our study did not find a significant change in nutritional status after the 6-month DCS, and further research on participants’ dietary intake and eating behaviors are therefore necessary to determine more precisely the beneficial effects of DCS on nutritional status among elderly people with dementia or disability.

In Taiwan, caregivers are usually family members (spouses or children) or other individuals (e.g., foreign domestic workers) and they provide the majority of the care that elderly patients receive [36]. Because the physical and psychological burden of caring for an older adult is considerable, many family caregivers develop psychological illnesses and experience depression, which may result in caregivers taking time off work for long periods of time [37]. According to previous systemic reviews, it has been shown that DCS can provide family caregivers with support, and this in turn has positive effects on the relationship between persons with dementia and their family caregivers [5, 38]. Our preliminary result supports previous studies showing that DCS can decrease family caregivers’ exposure to primary stressors, and may also reduce the burden of care by improving quality of life.

There were several limitations in this study. First, our study had a small sample size and the study data were collected in a period of less than 6 months. Second, we did not examine the effects of specific interventions in the DCS programs on the participants’ well-being. It is possible that some of the activities may not have been optimal for all of the older people in the study. Third, there was no control group in this study, and thus it was not possible to determine whether the mitigation of progressive impairment was a result of DCS.Fourth, the costs of DCS for individuals with dementia are estimated from $500 to $700 dollars per month according to the stage of dementia, but a cost-effectiveness analysis was not conducted in the present study. Finally, some other potential factors affecting cognitive and physical functional decline, such as medications, and laboratory values, were not examined in the current study. Further longitudinal analyses with larger numbers of participants are required to establish whether DCS can improve cognition and physical functions in disabled older people.

## Conclusions

Our study showed that multiple non‐pharmacological activities of DCS over a 6-month period was associated with short-term maintainance of physical and mental functions in elderly people with dementia or disability in a day care center. Besides, the family caregivers’ burden was reduced. To examine the individual effectiveness of various programs in DCS, further randomized controlled studies with a large sample size and longer follow-up time are necessary.

## Data Availability

The datasets used and/or analyzed during the current study are available and can be obtained from the corresponding author on reasonable request.

## Abbreviations

ACCI: Age-adjusted Charlson Comorbidity Index
ADL: Activities of daily living
CDR: Clinical dementia rating
CFS: Clinical frailty scale
CGA: Comprehensive geriatric assessment
DCS: Day care service
EQ-5D-3L: 3-Level version of European Quality of life-5 dimensions
EQ-VAS: EQ visual analogue scale
GDS-5: Five-item Geriatric Depression Scale
IADL: Instrumental Activities of Daily Living
IQR: Interquartile range
LTC: Long-term care
MMSE: Mini-mental state examination
MNA-SF: Mini -Nutritional Assessment-Short Form
